# Association of Reduced Folate Carrier-1 (RFC-1) Polymorphisms with Ischemic Stroke and Silent Brain Infarction

**DOI:** 10.1371/journal.pone.0115295

**Published:** 2015-02-06

**Authors:** Yunkyung Cho, Jung O Kim, Jeong Han Lee, Hye Mi Park, Young Joo Jeon, Seung Hun Oh, Jinkun Bae, Young Seok Park, Ok Joon Kim, Nam Keun Kim

**Affiliations:** 1 Department of Internal Medicine, CHA Gangnam Medical Center, School of Medicine, CHA University, Seoul 135–081, South Korea; 2 Institute for Clinical Research, CHA Bundang Medical Center, School of Medicine, CHA University, Seongnam 463–712, South Korea; 3 Department of Emergency Medicine, CHA Bundang Medical Center, School of Medicine, CHA University, Seongnam 463–712, South Korea; 4 Department of Neurology, CHA Bundang Medical Center, School of Medicine, CHA University, Seongnam 463–712, South Korea; 5 Department of Neurosurgery, Chungbuk National University Hospital, Chungbuk National University, Cheongju 361–711, South Korea; CSIR-INSTITUTE OF GENOMICS AND INTEGRATIVE BIOLOGY, INDIA

## Abstract

Stroke is the second leading cause of death in the world and in South Korea. Ischemic stroke and silent brain infarction (SBI) are complex, multifactorial diseases influenced by multiple genetic and environmental factors. Moderately elevated plasma homocysteine levels are a major risk factor for vascular diseases, including stroke and SBI. Folate and vitamin B12 are important regulators of homocysteine metabolism. Reduced folate carrier (RFC), a bidirectional anion exchanger, mediates folate delivery to a variety of cells. We selected three known *RFC-1* polymorphisms (-43C>T, 80A>G, 696T>C) and investigated their relationship to cerebral infarction in the Korean population. We used the polymerase chain reaction-restriction fragment length polymorphism (PCR-RFLP) method to analyze associations between the three *RFC-1* polymorphisms, disease status, and folate and homocysteine levels in 584 ischemic stroke patients, 353 SBI patients, and 505 control subjects. The frequencies of the *RFC-1* -43TT, 80GG, and 696CC genotypes differed significantly between the stroke and control groups. The *RFC-1* 80A>G substitution was also associated with small artery occlusion and SBI. In a gene-environment analysis, the *RFC-1* -43C>T, 80A>G, and 696T>C polymorphisms in the ischemic stroke group had combined effects with all environmental factors. In summary, we found that the *RFC-1* -43C>T, 80A>G, and 696T>C polymorphisms may be risk factors for ischemic stroke.

## Introduction

Stroke is the second leading cause of death in the world and in South Korea [1, 2]. Approximately 80% of strokes are ischemic in origin [3, 4]. Ischemic stroke is a complex, multifactorial disease influenced by multiple genetic and environmental factors [5, 6]. Stroke risk is associated with various biological pathways, including the homocysteine metabolism, lipid metabolism, and hemostasis [7–9]. Silent brain infarction (SBI) is an asymptomatic infarction that is often incidentally detected via computed tomography (CT) scans or magnetic resonance imaging (MRI) in subjects with no history of stroke. The presence of SBI is an independent risk factor for the development of symptomatic infarction [10–14].

Moderately elevated plasma homocysteine levels are a major risk factor for vascular diseases, including stroke and SBI [7, 15–19]. A meta-analysis of prospective observational studies showed that a 25% lower plasma homocysteine level was associated with an 11% lower risk of ischemic heart disease and a 19% lower risk of stroke [20]. Folate and vitamin B12 are important regulators of homocysteine metabolism, and studies show an inverse relationship between folate intake and plasma homocysteine [21]. Several folate metabolism enzyme polymorphisms are associated with elevated plasma homocysteine levels [7, 22–25]. Taken together, these observations suggest that the regulation of plasma homocysteine and folate levels is associated with stroke and SBI risk.

Folate supplies the carbon group needed to methylate homocysteine and form methionine, with vitamin B12 acting as a cofactor [26]. The transport of folate and its analogs into mammalian cells occurs by carrier-mediated, as well as receptor-mediated, mechanisms. The reduced-folate carrier (RFC) protein is a bidirectional anion exchanger that mediates folate delivery into a variety of cells [27, 28]. In previous studies, the human gene *RFC-1*, also known as *SLC19A1*, has been cloned, and the primary structure of the protein has been resolved [29, 30].

*RFC-1* has a number of single nucleotide polymorphisms (SNPs). We selected three (-43C>T, 80A>G, and 696T>C) and investigated their relationship to ischemic stroke and SBI in Korean patients. In addition, we studied the association between the selected *RFC-1* polymorphisms and homocysteine and folate levels in patients and control subjects.

## Materials and Methods

### Study subjects

This study, including the consent procedure, was reviewed and approved by the institutional review board (IRB) of CHA Bundang Medical Center in October 2009, and written informed consent was obtained from all participants. We recruited 584 consecutive ischemic stroke patients referred by the Department of Neurology at CHA Bundang Medical Center from March 2004 to February 2010. All ischemic stroke patients suffered from rapidly developing clinical symptoms and signs of focal and/or global loss of brain function and displayed evidence of cerebral infarction in clinically relevant areas of the brain, according to MRI and electrocardiography. Based on clinical manifestations and neuroimaging data, two neurologists used the Trial of Org10172 in Acute Stroke Treatment criteria to classify ischemic strokes into four etiologic subtypes: small artery occlusion (SAO), large artery occlusion (LAO), cardiac embolism (CE), and undetermined (UD) [31]. Single and multiple (≥2) lesion SAOs were distinguished using brain MRI scans. The sizes and sites of cerebral infarctions were documented only with MRI.

We recruited 353 patients diagnosed with SBI by two independent neurologists after MRI scans and electrocardiography at the CHA Bundang Medical Center. All SBI patients had: 1) spotted areas ≥3 mm in diameter and supplied by deep perforating arteries, showing high intensity in the T2 and fluid attenuated inversion recovery images and low intensity in the T1 image, 2) the absence of neurological signs and symptoms that could be explained by the lesions observed by MRI, and 3) no clinical history of strokes including transient ischemic attacks. We did not consider small punctate hyperintensities (1–2 mm in diameter) in this study because they likely represented dilated perivascular spaces.

We recruited 505 control subjects matched by sex and age within 5 years to the ischemic stroke and SBI patients. Control subjects received health examinations at our hospitals, including biochemistry testing, an electrocardiogram, and a brain MRI, during the recruitment period. None of the control subjects had a recent history of cerebrovascular disease or myocardial infarction. We confirm that all data underlying the findings are fully available without restriction (S1 Table in S1 File). All relevant data are within the paper and its Supporting Information files (S1 Table in S1 File and S2-S13 Tables in S2 File).

### Genotyping *RFC-1* polymorphisms

We obtained DNA from subject blood samples using the G-DEX blood extraction kit (iNtRON Biotechnology, Inc., Seongnam, South Korea). We performed a bioinformatic search of the HapMap database (http://www.hapmap.org) to identify three known SNPs in *RFC-1*: –43C>T (rs1131596), 80A>G (rs1051266), and 696T>C (rs12659). We screened study participants for these polymorphisms using the polymerase chain reaction-restriction fragment length polymorphism (PCR-RFLP) method [32].

### Estimation of homocysteine and folate levels

We collected plasma samples to measure homocysteine and folate levels within 48 hours of stroke onset or SBI detection. Twelve hours after the patient's last meal, we collected whole blood in a tube containing anticoagulant. We centrifuged the tube for 15 min at 1000 × *g* to separate the plasma. We then measured plasma homocysteine concentrations using a fluorescent polarizing immunoassay with the IMx system (Abbott Laboratories, Chicago, IL, USA) and plasma folate concentrations with a radioassay kit (ACS 180; Bayer, Tarrytown, NY, USA).

### Statistical analysis

We estimated associations between the *RFC-1* genotypes and ischemic stroke and SBI by computing odds ratios (ORs) and 95% confidence intervals (CIs) and by performing Fisher’s exact test. We determined the adjusted odds ratios (AORs) for the *RFC-1* polymorphisms by performing a multiple logistic regression analysis that included sex, age, diabetes mellitus, hypertension, hyperlipidemia, and smoking variables. We also performed an analysis of stroke subgroups, stratified according to the size of the occluded vessel. We compared mean homocysteine concentrations among different genotypes using one-way analysis of variance. We tested multiple hypotheses using the Benjamini-Hochberg procedure to control the false discovery rate in unconditional logistic regression analysis [33]. Calculating the false discovery rate addresses the effect of multiple comparisons on the overall experimental error rate by estimating the proportion of false positives among the data. We accepted statistical significance for all hypothesis tests at the *P*<0.05 level. StatsDirect Statistical Software (version 2.4.4; StatsDirect Ltd, Altrincham, UK) was used to calculate AORs and 95% CIs. We estimated haplotype frequencies for multiple loci using the expectation-maximization algorithm and SNPAlyze (version 5.1; DYNACOM Co., Ltd., Yokohama, Japan).

## Results

Table 1 shows the clinical characteristics of the case and control groups. Both the ischemic stroke and SBI groups had significantly higher plasma homocysteine and folate concentrations than controls. Table 2 shows a comparison of *RFC-1* -43C>T, 80A>G, and 696T>C genotype frequencies in the ischemic stroke, SBI, and control groups. The frequencies of the -43TT genotype, the 80GG genotype, and the 696CC genotype were significantly higher in the ischemic stroke group than in the control group. Initially, the frequency of the *RFC-1* 80AG+GG genotypes appeared to be significantly higher in the SBI group than the control group; however, after correcting for multiple testing, this result was no longer significant. By contrast, the *RFC-1* -43C>T, 80A>G, and 696T>C polymorphisms were associated with significantly increased odds of ischemic stroke, even after correcting for multiple testing. Thus, there was a significantly higher prevalence of *RFC-1*–43/80/696 minor alleles in ischemic stroke patients than in controls. Logistic regression demonstrated that the frequency of the *RFC-1* minor alleles (-43T, 80G, and 696C) was significantly different between ischemic stroke patients and controls (*P*<0.05; Table 2).

**Table 1:** Baseline characteristics of ischemic stroke patients, silent brain infarction (SBI) patients, and control subjects.

Characteristics	Control (%)	Ischemic stroke (%)	*P* ^a^	SBI (%)	*P* ^b^
N	505	584		353	
Male (%)	262 (51.9)	328 (56.2)	0.157	163 (46.2)	0.100
Age (year)	63.87 ± 10.59	64.61 ± 11.69	0.279	64.13 ± 11.51	0.734
tHcy (μmol/L, n)^c^	10.19 ± 3.963 (500)	11.56 ± 6.767 (584)	<0.0001	11.64 ± 6.466 (351)	<0.0001
Between-person CV, %	38.9	58.5		55.5	
Folate (ng/ml, n)^c^	9.024 ± 8.235 (399)	7.007 ± 6.457 (579)	<0.0001	8.958 ± 5.993 (344)	0.903
Between-person CV, %	91.3	92.2		66.9	
Hypertension (%)	245 (48.5)	371 (63.5)	<0.0001	182 (51.6)	0.380
Diabetes mellitus (%)	81 (16.0)	162 (27.7)	<0.0001	51 (14.4)	0.525
Hyperlipidemia (%)	108 (21.4)	166 (28.4)	0.008	96 (27.2)	0.049
Smoking (%)	142 (28.1)	256 (43.8)	<0.0001	-	-
BUN (mg/dl)^c^	16.05 ± 5.43 (475)	16.39 ± 6.66 (583)	0.382	16.31 ± 6.01 (339)	0.519
Urate (mg/dl)^c^	4.76 ± 1.43 (455)	4.77 ± 1.59 (583)	0.931	4.71 ± 1.60 (324)	0.607
Platelet (103 cells/μl)^c^	235.38 ± 73.39 (473)	247.88 ± 130.42 (584)	0.063	238.83 ± 71.84 (336)	0.506
Prothrombin time (sec)^c^	12.02 ± 1.31 (346)	11.84 ± 1.04 (578)	0.024	12.23 ± 1.51 (223)	0.079
aPTT (sec)^c^	33.23 ± 17.10 (346)	30.89 ± 4.65 (578)	0.002	33.69 ± 8.26 (231)	0.703
Fibrinogen (mg/dl)^c^	129.60 ± 100.72 (122)	422.86 ± 129.50 (560)	<0.0001	395.85 ± 124.03 (115)	<0.0001
Antithrombin III (%)^c^	342.43 ± 148.00 (119)	92.01 ± 17.62 (560)	<0.0001	98.05 ± 17.46 (117)	<0.0001

CV, coefficient of variation; tHcy, plasma total homocysteine; aPTT, activated partial thromboplastin time; BUN, blood urea nitrogen. ^a^ *P*<0.05 indicates a significant difference between ischemic stroke patients and controls.

^b^ *P*<0.05 indicates a significant difference between SBI patients and controls.

^c^ *P*-values obtained from Mann-Whitney non-parametric test.

**Table 2:** Comparison of genotype frequencies and AOR values for the *RFC-1* -43C>T, 80A>G, and 696T>C polymorphisms in the ischemic stroke patients, silent brain infarction (SBI) patients, and control subjects.

		Ischemic stroke				Silent brain infarction			
Genotype	Control (%) n = 505	Case (%) n = 584	AOR (95% CI)	*P* ^a^	*P* ^c^	Case (%) n = 353	AOR (95% CI)	*P* ^b^	*P* ^c^
*RFC-1* -43C>T									
CC	146 (28.9)	148 (25.3)	1.000 (Reference)			103 (29.2)	1.000 (Reference)		
CT	265 (52.5)	303 (51.9)	1.167 (0.868–1.567)	0.306	0.306	185 (52.4)	0.990 (0.723–1.355)	1.000	1.000
TT	94 (18.6)	133 (22.8)	1.475 (1.024–2.124)	0.037	0.037	65 (18.4)	0.980 (0.654–1.469)	1.000	1.000
CC vs. CT+TT (Dominant)			1.252 (0.947–1.657)	0.115	0.115		0.987 (0.732–1.332)	0.939	0.939
CC+CT vs. TT (Recessive)			1.316 (0.967–1.791)	0.081	0.084		0.987 (0.695–1.401)	1.000	1.000
C allele	557 (55.1)	599 (51.3)	1.00 (Reference)			391 (55.4)	1.00 (Reference)		
T allele	453 (44.9)	569 (48.7)	1.196 (1.003–1.426)	0.046	0.046	315 (44.6)	1.010 (0.831–1.227)	0.924	0.924
*RFC-1* 80A>G									
AA	172 (34.1)	172 (29.4)	1.000 (Reference)			97 (27.5)	1.000 (Reference)		
AG	240 (47.5)	279 (47.8)	1.190 (0.895–1.583)	0.238	0.306	184 (52.1)	1.364 (0.994–1.873)	0.055	0.165
GG	93 (18.4)	133 (22.8)	1.459 (1.026–2.075)	0.035	0.037	72 (20.4)	1.422 (0.952–2.123)	0.085	0.255
AA vs. AG+GG (Dominant)			1.267 (0.970–1.655)	0.082	0.115		1.384 (1.025–1.867)	0.034	0.102
AA+AG vs. GG (Recessive)			1.312 (0.964–1.785)	0.084	0.084		1.166 (0.825–1.647)	0.384	0.746
A allele	584 (57.8)	623 (53.3)	1.000 (Reference)			378 (53.5)	1.000 (Reference)		
G allele	426 (42.2)	545 (46.7)	1.213 (1.017–1.447)	0.032	0.046	328 (46.5)	1.207 (0.993–1.467)	0.059	0.177
*RFC-1* 696T>C									
TT	146 (28.9)	142 (24.3)	1.000 (Reference)			92 (26.1)	1.000 (Reference)		
TC	262 (51.9)	300 (51.4)	1.208 (0.897–1.627)	0.214	0.306	189 (53.5)	1.136 (0.822–1.571)	0.439	0.659
CC	97 (19.2)	142 (24.3)	1.549 (1.082–2.219)	0.017	0.037	72 (20.4)	1.218 (0.811–1.831)	0.342	0.513
TT vs. TC+CC (Dominant)			1.307 (0.986–1.731)	0.063	0.115		1.163 (0.855–1.583)	0.336	0.504
TT+TC vs. CC (Recessive)			1.373 (1.015–1.857)	0.040	0.084		1.127 (0.799–1.589)	0.497	0.746
T allele	554 (54.9)	584 (50.0)	1.000 (Reference)			373 (52.8)	1.000 (Reference)		
C allele	456 (45.1)	584 (50.0)	1.236 (1.037–1.474)	0.018	0.046	333 (47.2)	1.103 (0.909–1.340)	0.321	0.482

AOR, adjusted odds ratio; 95% CI, 95% confident interval.

^a^ *P*-values on the basis of risk factors such as age, gender, hypertension, hyperlipidemia, diabetes mellitus, and smoking.

^b^ *P*-values on the basis of risk factors such as age, gender, hypertension, hyperlipidemia, and diabetes mellitus.

^c^ False discovery rate-adjusted *P* value for multiple hypothesis testing using the Benjamini-Hochberg method.

When we stratified ischemic stroke data by the size of the occluded vessel, we found an association between all three *RFC-1* polymorphisms and both single and multiple SAOs (Table 3 and S2 Table in S2 File). However, we did not find significant associations between any of the *RFC-1* polymorphisms and the other ischemic stroke subtypes (i.e., LAO, CE, and UD; Table 3). The frequencies of the -43TT genotype, the 80GG genotype, and the 696CC genotype were also significantly higher in SAO patients than controls. Furthermore, the frequencies of dominant and recessive models presented significant *P*-values (<0.005) in SAO patients than controls. A comparison of *RFC-1* polymorphism genotype frequencies and AORs for stroke subtypes and patients with SBI is shown as S3 Table in S2 File. There were significant frequency differences for the—43C>T polymorphism between SAO patients and multiple SAO subtypes and patients with SBI.

**Table 3:** Comparison of genotype frequencies and AOR for the *RFC-1* -43C>T, 80A>G, and 696T>C polymorphisms between the ischemic stroke subgroups with small-artery occlusion, large-artery occlusion, cardio embolism, undetermined and control subjects.

		Small-artery occlusion				Large-artery occlusion				Cardio embolism				Undetermined			
Genotype	Control (%) n = 505	Case (%) n = 159	AOR (95%CI)	*P* ^a^	*P* ^b^	Case (%) n = 241	AOR (95%CI)	*P* ^a^	*P* ^b^	Case (%) n = 70	AOR (95%CI)	*P* ^a^	*P* ^b^	Case (%) n = 108	AOR (95%CI)	P	*P*†
*RFC-1* -43C>T																	
CC	146 (28.9)	37 (23.3)	1.000 (Reference)			64 (26.6)	1.000 (Reference)			17 (24.3)	1.000 (Reference)			29 (26.8)	1.000 (Reference)		
CT	265 (52.5)	77 (48.4)	1.239 (0.786–1.954)	0.357	0.357	128 (53.1)	1.146 (0.779–1.684)	0.489	0.489	36 (51.4)	1.105 (0.587–2.081)	0.757	0.890	57 (52.8)	1.219 (0.724–2.053)	0.456	0.459
TT	94 (18.6)	45 (28.3)	2.152 (1.266–3.659)	0.005	0.005	49 (20.3)	1.220 (0.751–1.982)	0.422	0.451	17 (24.3)	1.408 (0.666–2.978)	0.371	0.371	22 (20.4)	1.381 (0.719–2.655)	0.333	0.455
CC vs. CT+TT (Dominant)			1.472 (0.960–2.257)	0.076	0.076		1.164 (0.809–1.676)	0.414	0.414		1.204 (0.663–2.185)	0.542	0.792		1.248 (0.762–2.044)	0.379	0.388
CC+CT vs. TT (Recessive)			1.835 (1.197–2.814)	0.005	0.008		1.087 (0.722–1.636)	0.691	0.781		1.275 (0.696–2.335)	0.431	0.431		1.218 (0.703–2.110)	0.482	0.613
*RFC-1* 80A>G																	
AA	172 (34.1)	40 (25.2)	1.000 (Reference)			75 (31.1)	1.000 (Reference)			22 (31.4)	1.000 (Reference)			34 (31.5)	1.000 (Reference)		
AG	240 (47.5)	74 (46.5)	1.437 (0.920–2.244)	0.113	0.327	118 (49.0)	1.176 (0.811–1.705)	0.392	0.489	30 (42.9)	0.941 (0.518–1.711)	0.842	0.890	52 (48.1)	1.208 (0.732–1.993)	0.459	0.459
GG	93 (18.4)	45 (28.3)	2.358 (1.397–3.981)	0.001	0.003	48 (19.9)	1.197 (0.750–1.911)	0.451	0.451	18 (25.7)	1.404 (0.705–2.799)	0.335	0.371	22 (20.4)	1.339 (0.713–2.512)	0.364	0.455
AA vs. AG+GG (Dominant)			1.677 (1.108–2.540)	0.015	0.045		1.171 (0.828–1.658)	0.372	0.414		1.077 (0.621–1.867)	0.792	0.792		1.230 (0.769–1.968)	0.388	0.388
AA+AG vs. GG (Recessive)			1.854 (1.208–2.847)	0.005	0.008		1.060 (0.703–1.598)	0.781	0.781		1.409 (0.778–2.553)	0.258	0.387		1.203 (0.695–2.082)	0.510	0.613
*RFC-1* 696T>C																	
TT	146 (28.9)	35 (22.0)	1.000 (Reference)			60 (24.9)	1.000 (Reference)			17 (24.3)	1.000 (Reference)			29 (26.8)	1.000 (Reference)		
TC	262 (51.9)	79 (49.7)	1.336 (0.843–2.119)	0.218	0.327	126 (52.3)	1.203 (0.813–1.780)	0.356	0.489	33 (47.1)	1.046 (0.552–1.981)	0.890	0.890	57 (52.8)	1.246 (0.739–2.101)	0.408	0.459
CC	97 (19.2)	45 (28.3)	2.160 (1.267–3.683)	0.005	0.005	55 (22.8)	1.355 (0.844–2.174)	0.209	0.451	20 (28.6)	1.564 (0.757–3.231)	0.227	0.371	22 (20.4)	1.281 (0.669–4.450)	0.455	0.455
TT vs. TC+CC (Dominant)			1.559 (1.010–2.406)	0.045	0.068		1.249 (0.863–1.806)	0.238	0.414		1.206 (0.665–2.188)	0.538	0.792		1.248 (0.762–2.045)	0.378	0.388
TT+TC vs. CC (Recessive)			1.747 (1.142–2.673)	0.010	0.010		1.195 (0.805–1.775)	0.376	0.781		1.512 (0.847–2.697)	0.162	0.387		1.151 (0.667–1.988)	0.613	0.613

AOR, adjusted odds ratio; 95% CI, 95% confident interval.

^a^ *P*-values on the basis of risk factors such as age, gender, hypertension, hyperlipidemia, diabetes mellitus, and smoking.

^b^ False discovery rate-adjusted *P* value for multiple hypothesis testing using the Benjamini-Hochberg method.

We constructed haplotypes for the three *RFC-1* polymorphisms (Table 4). The frequencies of several haplotypes differed significantly between SAO patients and controls (S3 and S4 Tables in S2 File). For example, compared to controls, the frequency of the C-A-T (-43/80/696), C-A (-43/80), and A-T (80/696) haplotypes was significantly lower in (1) all stroke patients and (2) SAO patients only. By contrast, compared to controls, the frequency of the C-G-T (-43/80/696) and C-G (-43/80) haplotypes was significantly higher in (1) all stroke patients and (2) SAO patients only. We also found different haplotype frequencies between SBI patients and controls. For example, the frequency of the T-A-C, T-G-T (-43/80/696), and T-A (-43/80) haplotypes was significantly lower in SBI patients than controls. Furthermore, the frequency of the T-G-T (-43/80/696) haplotype was significantly lower in all stroke patients than controls.

**Table 4:** The haplotype analysis of the *RFC-1* -43C>T, 80A>G, and 696T>C polymorphisms among the ischemic stroke patients, silent brain infarction (SBI) patients, and control subjects.

		Ischemic stroke patients						
Haplotype	Control	Stroke	SAO		LAO	CE	UD	SBI
*RFC-1–*43/80/696								
C-A-T	0.5283	0.4722 ^*^		0.4326 ^**^	0.4861	0.4486	0.5087	0.5183
C-A-C	0.0099	0.0099		0.0062	0.0141	0.0211	0.0000	0.0128
C-G-T	0.0051	0.0199 ^**^		0.0264 ^**^	0.0199	0.0225	0.0096	0.0085
C-G-C	0.0082	0.0108		0.0096	0.0109	0.0078	0.0141	0.0142
T-A-T	0.0080	0.0070		0.0063	0.0043	0.0074	0.0141	0.0015
T-A-C	0.0320	0.0442		0.0392	0.0515	0.0514	0.0327	0.0029 ^**^
T-G-T	0.0071	0.0009 ^*^		0.0033	0.0000	0.0000	0.0000	0.0000 ^*^
T-G-C	0.4014	0.4350		0.4764 ^*^	0.4131	0.4411	0.4208	0.4418
Overall^a^		0.1751		0.2744	0.3208	0.4227	0.7468	0.0419
*RFC-1–*43/80								
C-A	0.5381	0.4821 ^*^		0.4386 ^**^	0.5003	0.4697	0.5080	0.5311
C-G	0.0134	0.0307 ^*^		0.0362 ^*^	0.0308 ^*^	0.0303	0.0244	0.0228
T-A	0.0402	0.0513		0.0457	0.0557	0.0588	0.0475	0.0044 ^**^
T-G	0.4084	0.4359		0.4795 ^*^	0.4131	0.4412	0.4201	0.4418
*RFC-1* 80/696								
A-T	0.5361	0.4792 ^*^		0.4389 ^**^	0.4903	0.4556	0.5228	0.5197
A-C	0.0421	0.0542		0.0454	0.0657	0.0729	0.0327	0.0157 ^**^
G-T	0.0124	0.0208		0.0297	0.0200	0.0229	0.0096	0.0086
G-C	0.4094	0.4458		0.4861 ^*^	0.4240	0.4485	0.4349	0.4559
*RFC-1–*43/696								
C-T	0.5334	0.4921		0.4590	0.5061	0.4712	0.5183	0.5269
C-C	0.0181	0.0207		0.0159	0.0250	0.0288	0.0141	0.0270
T-T	0.0151	0.0079		0.0096	0.0043	0.0074	0.0141	0.0015 ^**^
T-C	0.4334	0.4793 ^*^		0.5156 ^*^	0.4646	0.4926	0.4535	0.4447

SAO, small artery occlusion; LAO, large artery occlusion; CE, cardioembolism; UD, undetermined.

^*^ *P*<0.05

^**^ *P*<0.01

*P*-value of each haplotype was calculated using two-sided chi-square test, with reference to all other haplotypes.

To further elucidate the genetic etiology of ischemic stroke and SBI, we examined the association between polymorphisms in ischemic stroke and SBI patients and various environmental factors, including homocysteine and folate levels, hypertension, diabetes mellitus, hyperlipidemia, and smoking (Table 5 and S5 Table in S2 File). We used the upper 15^th^ percentile of homocysteine levels and the lower 15^th^ percentile of folate levels as cut-off values in the analysis. Homocysteine cut-off values were 14.0 μmol/L in stroke patients and 13.61 μmol/L in SBI patients. Folate cut-off values were 3.32 ng/ml in stroke patients and 4.45 ng/ml in SBI patients. We found associations between each of the three polymorphisms and hyperhomocysteinemia and folate deficiency in ischemic stroke and SBI patients (Table 5).

**Table 5:** Combined effects of genotype and homocysteine and folate levels upon ischemic stroke and silent brain infarction (SBI) risk.

		tHcy≤14.00 μmol/L	tHcy>14.00 μmol/L	Folate>3.32 ng/ml	Folate≤3.32 ng/ml
Stroke	Genotype	AOR (95% CI) ^a^	AOR (95% CI) ^a^	AOR (95% CI) ^a^	AOR (95% CI) ^a^
	*RFC-1*–43CC	1.000 (reference)	2.062 (1.004–4.236)	1.000 (reference)	3.121 (1.353–7.198)
	*RFC-1*–43CT+TT	1.288 (0.952–1.743)	2.158 (1.315–3.541)	1.343 (0.978–1.845)	5.071 (2.686–9.575)
	*RFC-1* 80AA	1.000 (reference)	2.893 (1.443–5.797)	1.000 (reference)	3.424 (1.498–7.829)
	*RFC-1* 80AG+GG	1.374 (1.029–1.834)	2.171 (1.328–3.549)	1.347 (0.997–1.819)	7.410 (3.660–15.00)
	*RFC-1* 696TT	1.000 (reference)	2.118 (1.060–4.481)	1.000 (reference)	3.938 (1.657–9.357)
	*RFC-1* 696TC+CC	1.373 (1.012–1.864)	2.271 (1.386–3.719)	1.449 (1.053–1.995)	4.968 (2.659–9.284)
		tHcy≤13.61 μmol/L	tHcy>13.61 μmol/L	Folate>4.45ng/ml	Folate≤4.45ng/ml
SBI	Genotype	AOR (95% CI) ^b^	AOR (95% CI) ^b^	AOR (95% CI) ^b^	AOR (95% CI) ^b^
	*RFC-1*–43CC	1.000 (reference)	2.127 (1.011–4.477)	1.000 (reference)	0.985 (0.460–2.109)
	*RFC-1*–43CT+TT	1.071 (0.768–1.492)	1.528 (0.905–2.579)	1.107 (0.784–1.563)	1.051 (0.597–1.850)
	*RFC-1* 80AA	1.000 (reference)	2.730 (1.320–5.643)	1.000 (reference)	1.215 (0.585–2.526)
	*RFC-1* 80AG+GG	1.562 (1.121–2.176)	2.060 (1.221–3.475)	1.595 (1.132–2.249)	1.598 (0.905–2.824)
	*RFC-1* 696TT	1.000 (reference)	2.103 (0.974–4.539)	1.000 (reference)	1.035 (0.464–2.310)
	*RFC-1* 696TC+CC	1.238 (0.883–1.738)	1.850 (1.094–3.128)	1.310 (0.922–1.863)	0.232 (0.702–2.163)

tHcy, total homocysteine; AOR, adjusted odds ratio; 95% CI, 95% confident interval.

^a^ AORs on the basis of risk factors such as age, gender, hypertension, hyperlipidemia, diabetes mellitus, and smoking.

^b^ AORs on the basis of risk factors such as age, gender, hypertension, hyperlipidemia, and diabetes mellitus.

The combined gene-environment analysis revealed many combined effects of genotype and clinical factors on ischemic stroke risk (S5 Table in S2 File). For example, the *RFC-1–*43, 80, and 696 polymorphisms showed combined effects in combination with all environmental factors in ischemic stroke patients. To detect any possible correlations between homocysteine and folate levels, we classified all subjects by their *RFC-1* genotype: wild type homozygous, heterozygous, and mutant homozygous (S6 Table in S2 File). We found significant inverse correlations between homocysteine and folate levels in heterozygous (*RFC-1*–43CT, 80AG, 696TC) individuals in the control and ischemic stroke groups and homozygous (*RFC-1* -43TT, 80GG, 696CC) individuals in the ischemic stroke, SBI, and control groups. In addition, we analyzed the correlation between homocysteine and folate levels after stratifying by sex, *RFC-1* genotype, and ischemic stroke/silent brain infarction/control status (S7 Table in S2 File). This analysis showed that a significant correlation between homocysteine and folate levels occurred more often in female groups than male groups. Interestingly, this correlation was present in all *RFC-1* mutant homozygote groups with SBI, for both sexes (S6 and S7 Tables in S2 File).

S8 and S9 Tables in S2 File show homocysteine and folate levels stratified by *RFC-1* genotype and disease condition. We analyzed a total of 1,442 individuals (584 stroke patients, 353 SBI patients, and 505 controls) from two different case-control samples according to date of recruitment (S10–S13 Tables in S2 File). In both samples, the *RFC-1* 80A>G polymorphism was significantly elevated among both SAO subtypes. This result suggests that the *RFC-1* 80A>G substitution is an important risk factor for stroke caused by SAO.

## Discussion

Folate is a crucial nutrient that supports important physiological functions, such as DNA synthesis, cell division, and substrate methylation. Folate is the strongest nutritional and pharmacological determinant of plasma homocysteine concentrations, which are associated with an increased risk of cardiovascular disease, though the relationship has not been proven to be causal [34]. Some evidence supports an independent protective effect for folate against vascular diseases. For example, several studies suggest that increased folate intake is associated with decreased risk of ischemic stroke and cardiovascular disease [35–38]. Regardless of the exact mechanism, folate metabolism is strongly associated with ischemic stroke and SBI.

Intracellular uptake of folate is mediated in part by RFC-1, which is encoded by the human solute carrier family 19, member 1 (*SLC19A1*) gene. RFC-1 is a high capacity, bidirectional transporter of 5-methyltetrahydrofolate and thiamine monophosphate. In addition to its role in folate uptake, RFC-1 plays a critical role in folate homeostasis in mammalian cells, where it is down-regulated in response to folate deficiency [39].

Human *RFC-1* has many polymorphic sites; above all, *RFC-1* 80A>G has been observed in association with disease by several research groups. In one Italian study, the *RFC-1* 80A>G substitution was elevated among children with neural tube defects, their mothers, and their fathers [40]. Another study on the same substitution in China found that among infants whose mothers did not use folate supplements during pregnancy, the risk of congenital heart disease was significantly higher for infants with the GG and AG genotypes than the AA genotype [41]. In yet another study, the A allele had a significant protective effect against thrombosis [42]. Several studies have also investigated the relationship between this polymorphism and various cancers; one found a significant association between *RFC-1* 80A>G and esophageal and gastric cancers in the Chinese population [43].

In this study, we found that individuals with the *RFC-1* 80A>G substitution have a higher risk of ischemic stroke and SBI. We found similar results for SAO. A previous report has shown that the homozygous AA genotype is associated with high plasma folate levels [44]. In addition, increased folate intake is associated with a decreased risk of ischemic stroke in men [35]. These previous results, together with our findings, suggest that the *RFC-1* 80G allele is related to low plasma folate levels. We found a relationship between *RFC-1* polymorphisms and hyperhomocysteinemia and folate deficiency in individuals with ischemic stroke as well as some heterozygous and mutant homozygous SBI groups. Our results suggest that the *RFC-1*–43, 80, and 696 polymorphisms affect plasma homocysteine and folate levels, albeit indirectly (S5 and S6 Tables in S2 File).

The *RFC-1* -43C>T and 696T>C polymorphisms are not as well studied as the 80A>G polymorphism. One study found that the *RFC-1* -43C>T polymorphism is associated with RFC-1 expression in rheumatoid arthritis patients receiving methotrexate treatment [45]. Another study found that the *RFC-1* 696T>C polymorphism is significantly more common in spontaneously aborted embryos than control children [46]. The *RFC-1* 696T>C polymorphism, especially the homozygous C genotype, has also been associated with increased risk of lung cancer [47].

We found a significant association between the *RFC-1*–43 and 696 polymorphisms and risk of ischemic stroke. As was true of the *RFC-1* 80 polymorphism, we found associations between the *RFC-1*–43 and 696 polymorphisms and SAO. We did not, however, find a relationship between the *RFC-1*–43 and 696 polymorphisms and SBI, or between the polymorphisms and plasma folate and homocysteine levels.

The pathophysiological aspects of SBI and single SAOs are quite similar. Thus, we expect that the underlying genetics are also similar. As shown in S5 Table in S2 File, there were significant differences in the frequency of the—43C>T polymorphism between SBI and SAO cases and SBI and multiple SAO subtypes. However, –43C>T genotype frequency was not different between the SBI and single SAO groups. These results suggest that pathophysiological mechanisms differ between SBI and multiple SAOs and are similar to those from a study on *VEGF* polymorphisms [48].

The three polymorphisms we studied did not have a direct relationship with plasma homocysteine and folate levels. In a separate analysis, we analyzed correlations between homocysteine and folate levels in the control, ischemic stroke, and SBI groups. We found significant inverse correlations between homocysteine and folate levels in *RFC-1* heterozygous (*RFC-1*–43CT, 80AG, 696TC) and mutant homozygous (*RFC-1* -43TT, 80GG, 696CC) individuals in the control, ischemic stroke, and SBI groups; individuals heterozygous or mutant homozygous for the *RFC-1*–43, 80, and 696 substitutions tended to have high homocysteine and low folate levels. Hyperhomocysteinemia is a known risk factor for ischemic stroke and SBI [7, 15–20]. In addition, folate is used to treat hyperhomocysteinemia [21, 49].

Previous reports, combined with our findings, suggest that the three *RFC-1* polymorphisms work together to affect plasma levels of homocysteine and folate; these levels are related to risk of ischemic stroke and SBI. We performed a genotype analysis using upper 15^th^ percentile cut-off values for homocysteine and lower 15^th^ percentile cut-off values for folate. We found a relationship between *RFC-1* polymorphisms and hyperhomocysteinemia and folate deficiency in individuals with ischemic stroke as well as some heterozygous and mutant homozygous SBI groups. Our results suggest that the *RFC-1*–43, 80, and 696 polymorphisms affect plasma homocysteine and folate levels, albeit indirectly.

Our study suggests that *RFC-1* minor alleles (-43/80/696) are related to increased ischemic stroke risk (Table 2). In addition, our haplotype analysis revealed that the frequencies of several *RFC-1* haplotypes were significantly different between control subjects and patients with ischemic stroke (SAOs) and SBI. For example, C-G-T (-43/80/696) and C-G (-43/80) haplotype frequencies were higher in ischemic stroke patients than in control subjects. On the other hand, C-A-T (-43/80/696) and C-A (-43/80) haplotype frequencies were lower in ischemic stroke subjects than in control subjects. These results appear to be driven by the substitution of the G allele for the A allele at *RFC-1* 80, corresponding to the results of our genotype frequency analysis and lending further support to the hypothesis that the *RFC-1* 80 G allele is associated with ischemic stroke risk.

The present study has several limitations. First, it is not yet clear which genetic polymorphisms predict phenotypes associated with ischemic stroke and SBI. Second, our results cannot be extrapolated to other races, because interethnic variability in the frequency of stroke subtypes and genotypes may produce different results. Third, this was a hospital-based case–control study with relatively small stroke subtype sample sizes. Therefore, additional studies involving different races, ethnic groups, or samples of populations with homogeneous origins are needed to confirm our results.

In summary, we identified a relationship between the *RFC-1* -43C>T, 80A>G, and 696T>C polymorphisms and ischemic stroke. Certain *RFC-1* haplotypes also had a significant association with ischemic stroke. In addition, we found a relationship between *RFC-1* polymorphisms and hyperhomocysteinemia and folate deficiency. These findings suggest that the *RFC-1* 80G allele may be a useful marker for evaluating ischemic stroke risk. Furthermore, we propose that all three of the *RFC-1* polymorphisms that we examined are genetic determinants for ischemic stroke risk in the Korean population. Additional studies on the biological functions of RFC-1 are needed to fully understand the role of *RFC-1* polymorphisms in controlling plasma homocysteine and folate levels in ischemic stroke and SBI patients.

## Supporting Information

S1 File. (XLS)

S2 File. (PDF)
