# Supplementary material for: Association of Reduced Folate Carrier-1 (RFC-1) Polymorphisms with Ischemic Stroke and Silent Brain Infarction
Source: PLoS One. 2015 Feb 6;10(2):e0115295. doi: 10.1371/journal.pone.0115295 (PMC4319782; doi:10.1371/journal.pone.0115295)
Supplement: S2 File. — (PDF) [file pone.0115295.s002.pdf]

Table S2. AOR values of single and multiple-small artery occlusion prevalence among *RFC-1* genotypes in samples

Genotype	Control (%)	Single small-artery occlusion				Multiple small-artery occlusion			
		Case (%)	AOR (95% CI)[a]	*P*	*P*[b]	Case (%)	AOR (95% CI)[a]	*P*	*P*[b]
	n=505	n=76				n=83			
***RFC-1* -43C>T**									
CC	146 (28.9)	17 (22.4)	1.000 (Reference)			20 (24.1)	1.000 (Reference)		
CT	265 (52.5)	40 (52.6)	1.441 (0.772-2.687)	0.251	0.251	37 (44.6)	1.099 (0.600-2.014)	0.758	0.758
TT	94 (18.6)	19 (25.0)	2.357 (1.200-5.053)	0.028	0.028	26 (31.3)	2.227 (1.135-4.368)	0.020	0.040
CC vs. CT+TT (Dominant)			1.632 (0.901-2.956)	0.106	0.106		1.398 (0.802-2.439)	0.237	0.240
CC+CT vs. TT (Recessive)			1.774 (0.973-3.234)	0.062	0.062		1.963 (1.154-3.338)	0.013	0.037
***RFC-1* 80A>G**									
AA	172 (34.1)	17 (22.4)	1.000 (Reference)			23 (27.7)	1.000 (Reference)		
AG	240 (47.5)	38 (50.0)	1.868 (0.993-3.515)	0.053	0.156	36 (43.4)	1.211 (0.680-2.154)	0.516	0.758
GG	93 (18.4)	21 (27.6)	3.252 (1.521-6.954)	0.002	0.006	24 (28.9)	1.998 (1.033-3.863)	0.040	0.040
AA vs. AG+GG (Dominant)			2.146 (1.183-3.892)	0.012	0.036		1.429 (0.842-2.427)	0.186	0.240
AA+AG vs. GG (Recessive)			2.005 (1.115-3.606)	0.020	0.060		1.782 (1.037-3.063)	0.037	0.037
***RFC-1* 696T>C**									
TT	146 (28.9)	15 (19.7)	1.000 (Reference)			20 (24.1)	1.000 (Reference)		
TC	262 (51.9)	41 (53.9)	1.710 (0.895-3.268)	0.104	0.156	38 (45.8)	1.158 (0.634-2.117)	0.633	0.758
CC	97 (19.2)	20 (26.4)	2.783 (1.277-6.063)	0.010	0.015	25 (30.1)	2.041 (1.039-4.009)	0.038	0.040
TT vs. TC+CC (Dominant)			1.934 (1.042-3.591)	0.037	0.056		1.396 (0.800-2.435)	0.240	0.240
TT+TC vs. CC (Recessive)			1.832 (1.012-3.314)	0.046	0.062		1.768 (1.038-3.012)	0.036	0.037

[a] Adjusted by age, gender, hypertension, diabetes mellitus, hyperlipidemia, and smoking.

[b] False positive discovery rate-adjusted *P*-value.

Table S3. OR values of *RFC-1* haplotypes among the ischemic stroke, ischemic-stroke subtype, silent brain infarction (SBI), and control subjects

Haplotypes	Ischemic stroke		SBI		Small-artery occlusion (SAOs)		Large-artery occlusion (LAOs)		Cardio embolism		Undetermined	
	OR (95% CI)	P[a]	OR (95% CI)	P[a]	OR (95% CI)	P[a]	OR (95% CI)	P[a]	OR (95% CI)	P[a]	OR (95% CI)	P[a]
***RFC-1* –43/80/696**												
C-A-T	0.799 (0.675-0.946)	0.010	0.959 (0.795-1.157)	0.667	0.683 (0.530-0.881)	0.004	0.841 (0.677-1.045)	0.121	0.729 (0.511-1.040)	0.087	0.925 (0.689-1.241)	0.653
C-A-C	1.038 (0.447-2.413)	0.898	1.186 (0.479-2.933)	0.817	0.633 (0.138-2.905)	0.742	1.474 (0.557-3.896)	0.441	2.190 (0.595-8.058)	0.204	0.220 (0.013-3.772)	0.225
C-G-T	4.038 (1.529-10.66)	0.004	1.583 (0.481-5.207)	0.546	5.187 (1.684-15.98)	0.004	4.258 (1.447-12.53)	0.009	4.401 (1.040-18.63)	0.062	1.879 (0.362-9.751)	0.357
C-G-C	1.410 (0.582-3.416)	0.586	1.988 (0.809-4.889)	0.172	1.193 (0.315-4.525)	0.731	1.313 (0.427-4.036)	0.767	0.901 (0.112-7.263)	1.000	1.764 (0.464-6.707)	0.421
T-A-T	0.864 (0.323-2.310)	0.968	0.163 (0.020-1.309)	0.087	0.793 (0.167-3.754)	1.000	0.522 (0.110-2.468)	0.515	0.901 (0.112-7.263)	1.000	1.764 (0.446-6.707)	0.421
T-A-C	1.424 (0.909-2.231)	0.150	0.080 (0.019-0.334)	<0.0001	1.199 (0.610-2.356)	0.592	1.672 (0.979-2.855)	0.061	1.609 (0.696-3.718)	0.313	1.024 (0.446-2.351)	1.000
T-G-T	0.123 (0.015-1.000)	0.048	0.087 (0.005-1.528)	0.022	0.452 (0.055-3.690)	0.688	0.139 (0.008-2.434)	0.104	0.476 (0.027-8.388)	1.000	0.309 (0.018-5.435)	0.614
T-G-C	1.150 (0.969-1.364)	0.119	1.187 (0.981-1.435)	0.081	1.351 (1.048-1.741)	0.023	1.050 (0.842-1.310)	0.693	1.187 (0.831-1.696)	0.359	1.088 (0.807-1.465)	0.594
***RFC-1* –43/80**												
C-A	0.800 (0.676-0.948)	0.011	0.970 (0.803-1.170)	0.773	0.668 (0.518-0.861)	0.002	0.860 (0.692-1.069)	0.184	0.767 (0.538-1.093)	0.149	0.893 (0.665-1.198)	0.454
C-G	2.262 (1.213-4.220)	0.013	1.707 (0.844-3.456)	0.151	2.790 (1.277-6.097)	0.017	2.285 (1.094-4.774)	0.028	2.092 (0.679-6.450)	0.261	1.686 (0.601-4.732)	0.357
T-A	1.280 (0.852-1.922)	0.276	0.093 (0.029-0.301)	<0.0001	1.170 (0.639-2.144)	0.632	1.402 (0.852-2.309)	0.186	1.432 (0.657-3.122)	0.370	1.147 (0.565-2.328)	0.707
T-G	1.121 (0.945-1.330)	0.204	1.153 (0.954-1.394)	0.146	1.329 (1.032-1.712)	0.032	1.021 (0.819-1.273)	0.866	1.154 (0.808-1.648)	0.464	1.057 (0.784-1.423)	0.761
***RFC-1* 80/696**												
A-T	0.799 (0.675-0.945)	0.010	0.937 (0.777-1.131)	0.502	0.682 (0.529-0.879)	0.003	0.832 (0.669-1.034)	0.097	0.730 (0.512-1.041)	0.087	0.951 (0.709-1.277)	0.764
A-C	1.282 (0.862-1.908)	0.259	0.327 (0.167-0.638)	0.0004	1.036 (0.559-1.919)	0.875	1.599 (0.998-2.562)	0.057	1.730 (0.849-3.526)	0.132	0.753 (0.334-1.698)	0.574
G-T	1.609 (0.815-3.177)	0.224	0.604 (0.228-1.596)	0.358	2.234 (0.946-5.277)	0.076	1.625 (0.707-3.733)	0.265	1.679 (0.472-5.970)	0.431	0.717 (0.161-3.201)	1.000
G-C	1.164 (0.982-1.380)	0.089	1.223 (1.012-1.478)	0.038	1.375 (1.067-1.771)	0.016	1.061 (0.851-1.322)	0.613	1.183 (0.829-1.688)	0.361	1.114 (0.828-1.499)	0.494
***RFC-1* –43/696**												
C-T	0.847 (0.716-1.003)	0.060	0.970 (0.804-1.171)	0.774	0.742 (0.576-0.955)	0.021	0.896 (0.721-1.113)	0.346	0.779 (0.547-1.111)	0.176	0.941 (0.701-1.263)	0.708
C-C	1.156 (0.624-2.143)	0.760	1.549 (0.820-2.929)	0.193	0.880 (0.324-2.391)	1.000	1.407 (0.672-2.946)	0.430	1.621 (0.540-4.862)	0.331	0.776 (0.227-2.660)	1.000
T-T	0.515 (0.224-1.182)	0.165	0.086 (0.011-0.657)	0.002	0.632 (0.182-2.197)	0.587	0.276 (0.063-1.214)	0.073	0.477 (0.063-3.643)	0.709	0.934 (0.268-3.257)	1.000
T-C	1.203 (1.015-1.425)	0.036	1.048 (0.868-1.267)	0.630	1.391 (1.080-1.791)	0.012	1.134 (0.912-1.410)	0.266	1.269 (0.891-1.808)	0.204	1.085 (0.807-1.457)	0.598

[a] Two-sided chi-square test, each haplotype compared with all other haplotypes.

Table S4. The haplotype analysis of the *RFC-1* -43C>T, 80A>G, and 696T>C polymorphisms among the single and multiple-small artery occlusion, and control subjects

Haplotype	Control	Ischemic stroke patients					
		Single SAO			Multiple SAO		
***RFC-1* –43/80/696**			OR (95% CI)	P^a		OR (95% CI)	P^a
C-A-T	0.528	0.426**	0.666 (0.472-0.939)	0.023	0.439*	0.700 (0.503-0.974)	0.036
C-A-C	0.010	0.005	0.662 (0.084-5.213)	1.000	0.006	0.606 (0.077-4.768)	1.000
C-G-T	0.005	0.035**	6.837 (1.955-23.91)	0.005	0.019	3.699 (0.875-15.63)	0.090
C-G-C	0.008	0.021	2.522 (0.661-9.615)	0.165	0.000	0.354 (0.020-6.170)	0.610
T-A-T	0.008	0.000	0.387 (0.022-6.738)	0.607	0.012	1.527 (0.321-7.259)	0.640
T-A-C	0.032	0.043	1.475 (0.639-3.405)	0.336	0.037	1.146 (0.472-2.785)	0.812
T-G-T	0.007	0.007	0.949 (0.116-7.771)	1.000	0.000	0.402 (0.023-7.073)	0.602
T-G-C	0.401	0.464	1.146 (0.812-1.618)	0.479	0.487	1.424 (1.024-1.979)	0.041
Overall [b]		0.070			0.466		
***RFC-1* –43/80**							
C-A	0.538	0.432*	0.660 (0.468-0.931)	0.019	0.445*	0.962 (0.497-0.962)	0.029
C-G	0.013	0.055**	3.952 (1.629-9.589)	0.005	0.019	1.309 (0.372-4.608)	0.722
T-A	0.040	0.042	0.971 (0.405-2.329)	1.000	0.049	1.197 (0.551-2.601)	0.674
T-G	0.408	0.471	1.306 (0.928-1.840)	0.134	0.487	1.383 (0.995-1.923)	0.062
***RFC-1* 80/696**							
A-T	0.536	0.425*	0.648 (0.459-0.914)	0.015	0.451	0.715 (0.514-0.994)	0.054
A-C	0.042	0.048	1.086 (0.479-2.460)	0.830	0.043	0.990 (0.438-2.240)	1.000
G-T	0.012	0.042*	3.152 (1.179-8.423)	0.029	0.019	1.412 (0.398-5.009)	0.484
G-C	0.409	0.485	1.371 (0.974-1.931)	0.078	0.487	1.377 (0.991-1.915)	0.062
***RFC-1* -43/696**							
C-T	0.533	0.460	0.746 (0.530-1.050)	0.098	0.458	0.738 (0.531-1.026)	0.078
C-C	0.018	0.027	1.489 (0.497-4.463)	0.517	0.006	0.334 (0.044-2.520)	0.502
T-T	0.015	0.007	0.439 (0.058-3.351)	0.710	0.012	0.809 (0.183-3.571)	1.000
T-C	0.433	0.506	1.341 (0.953-1.887)	0.097	0.524*	1.438 (1.035-1.999)	0.035

Two-sided chi-square test, each haplotype compared with all other haplotypes.

* $P<0.05$, ** $P<0.01$.

[a] *P*-value was calculated using the omnibus chi-square test.

[b] Two-sided chi-square test, each haplotype compared with all other haplotype.

Table S5. Ischemic stroke and silent brain infarction (SBI) risk by combinatorial effects between genotypes and environmental factors

	Variable	Without HTN	With HTN	Without DM	With DM	Without Hyprelipidemia	With Hyprelipidemia	Non-smoking	Smoking
Ischemic Stroke [a]	–43CC	1.000 (Reference)	1.667 (1.021-2.723)	1.000 (Reference)	1.419 (0.804-2.505)	1.000 (Reference)	1.492 (0.860-2.589)	1.000 (Reference)	1.763 (1.006-3.090)
	–43CT+TT	1.194 (0.772-1.846)	2.128 (1.387-3.265)	1.163 (0.847-1.597)	2.342 (1.519-3.610)	1.294 (0.933-1.795)	1.773 (1.182-2.660)	1.228 (0.861-1.750)	2.207 (1.400-3.477)
	80AA	1.000 (Reference)	1.887 (1.196-2.976)	1.000 (Reference)	1.515 (0.890-2.581)	1.000 (Reference)	1.679 (1.012-2.785)	1.000 (Reference)	1.476 (0.874-2.492)
	80AG+GG	1.275 (0.840-1.934)	2.211 (1.473-3.317)	1.203 (0.889-1.628)	2.370 (1.551-3.621)	1.361 (0.994-1.862)	1.837 (1.222-2.760)	1.160 (0.830-1.623)	2.283 (1.478-3.528)
	696TT	1.000 (Reference)	1.507 (0.919-2.472)	1.000 (Reference)	1.439 (0.805-2.571)	1.000 (Reference)	1.247 (0.704-2.209)	1.000 (Reference)	1.850 (1.048-3.268)
	696TC+CC	1.169 (0.754-1.813)	2.138 (1.389-3.290)	1.214 (0.883-1.668)	2.450 (1.588-3.781)	1.280 (0.924-1.774)	1.828 (1.223-2.731)	1.287 (0.901-1.836)	2.350 (1.498-3.687)
SBI [b]	–43CC	1.000 (Reference)	0.969 (0.578-1.626)	1.000 (Reference)	0.873 (0.449-1.695)	1.000 (Reference)	1.224 (0.674-2.221)	-	-
	–43CT+TT	0.943 (0.615-1.446)	1.013 (0.655-1.567)	0.982 (0.712-1.356)	0.781 (0.453-1.347)	0.953 (0.679-1.337)	1.318 (0.852-2.041)	-	-
	80AA	1.000 (Reference)	1.097 (0.663-1.816)	1.000 (Reference)	0.918 (0.476-1.771)	1.000 (Reference)	1.086 (0.607-1.943)	-	-
	80AG+GG	1.431 (0.934-2.192)	1.470 (0.952-2.270)	1.404 (1.019-1.933)	1.162 (0.676-1.995)	1.305 (0.932-1.827)	1.942 (1.248-3.020)	-	-
	696TT	1.000 (Reference)	0.871 (0.510-1.489)	1.000 (Reference)	0.976 (0.489-1.950)	1.000 (Reference)	1.117 (0.600-2.079)	-	-
	696TC+CC	1.043 (0.675-1.613)	1.138 (0.730-1.774)	1.182 (0.851-1.644)	0.933 (0.542-1.608)	1.112 (0.787-1.570)	1.572 (1.012-2.443)	-	-

HTN, hypertension; DM, diabetes mellitus.

[a] The adjusted odds ratio on the basis of risk factors such as age, gender, hypertension, hyperlipidemia, diabetes mellitus, and smoking.

[b] The adjusted odds ratio on the basis of risk factors such as age, gender, hypertension, hyperlipidemia, and diabetes mellitus.

Table S6. Correlation between homocysteine and folate levels among *RFC-1* –43C>T, 80A>G, 696T>C polymorphisms in ischemic stroke, silent brain infarction (SBI), and control subjects

	**Control (n=505)**	**Ischemic stroke (n=584)**	**SBI (n=353)**	**Control (n=505)**	**Ischemic stroke (n=584)**	**SBI (n=353)**	**Control (n=505)**	**Ischemic stroke (n=584)**	**SBI (n=353)**
		RFC-1 **–43CC genotype**			*RFC-1* **80AA genotype**			*RFC-1* **696TT genotype**	
tHcy (μmol/L)	9.984±3.249 (145)	11.280±6.655 (148)	11.330±4.308 (102)	9.902±3.124 (171)	11.510±6.604 (172)	11.400±4.387 (96)	10.050±3.276 (145)	11.590±6.965 (142)	11.350±4.357 (91)
Folate (ng/ml)	10.520±12.070 (122)	7.251±6.267 (147)	9.059±6.356 (100)	10.110±11.100 (144)	7.223±7.543 (170)	9.236±6.628 (94)	10.540±12.060 (122)	7.113±6.307 (141)	8.953±6.130 (89)
Correlation coefficient [a]	-0.114	-0.159	-0.165	-0.098	-0.145	-0.134	-0.115	-0.16	-0.229
P value	0.210	0.054	0.103	0.245	0.059	0.201	0.207	0.058	0.032
		RFC-1 **–43CT genotype**			*RFC-1* **80AG genotype**			*RFC-1* **696TC genotype**	
tHcy (μmol/L)	10.430±4.519 (262)	11.570±5.659 (303)	12.160±7.838 (185)	10.580±4.676 (237)	11.440±5.615 (279)	12.100±7.827 (184)	10.390±4.522 (259)	11.470±5.568 (300)	12.070±7.764 (189)
Folate (ng/ml)	8.217±5.928 (206)	6.923±6.698 (299)	9.024±6.114 (182)	8.152±6.323 (185)	6.981±5.891 (276)	8.987±6.049 (180)	8.239±5.989 (203)	7.080±6.757 (296)	9.192±6.315 (185)
Correlation coefficient [a]	-0.215	-0.188	-0.139	-0.213	-0.215	-0.141	-0.208	-0.192	-0.117
P value	0.002	0.001	0.061	0.004	0.0003	0.060	0.003	0.001	0.114
		RFC-1 **–43TT genotype**			*RFC-1* **80GG genotype**			*RFC-1* **696CC genotype**	
tHcy (μmol/L)	9.811±3.216 (93)	11.840±8.916 (133)	10.640±4.599 (64)	9.721±3.225 (92)	11.860±8.902 (133)	10.800±4.631 (71)	9.866±3.232 (96)	11.700±8.665 (142)	10.880±4.644 (71)
Folate (ng/ml)	8.800±4.982 (71)	6.925±6.146 (133)	8.601±5.026 (62)	9.097±4.974 (70)	6.782±6.109 (133)	8.511±4.908 (70)	8.669±4.865 (74)	6.748±5.985 (142)	8.347±4.869 (70)
Correlation coefficient [a]	-0.248	-0.184	-0.309	-0.275	-0.174	-0.34	-0.283	-0.18	-0.337
P value	0.037	0.035	0.015	0.021	0.045	0.004	0.015	0.032	0.005

[a] Correlation coefficient using the Pearson correlation analysis

Table S7. Correlation between homocysteine and folate levels among RFC-1 –43C>T, 80A>G, 696T>C polymorphisms in ischemic stroke, silent brain infarction (SBI), and control subjects based on sex.

***RFC-1* -43C>T**

Characteristics	Male Control (n=262)	Male Ischemic stroke (n=328)	Male SBI (n=163)	Female Control (n=243)	Female Ischemic stroke (n=256)	Female SBI (n=190)
***RFC-1* –43CC genotype**						
tHcy (µmol/L)	10.186±2.974	16.995±12.842	12.130±3.686	9.810±3.478	14.725±11.712	10.698±4.677
Folate (ng/ml)	7.755±6.594	6.782±6.821	8.693±7.133	12.433±14.469	7.913±5.376	9.335±5.753
Correlation coefficient [a]	0.115	-0.078	-0.106	-0.172	0.033	-0.205
P value	0.426	0.474	0.506	0.149	0.802	0.125
***RFC-1* –43CT genotype**						
tHcy (µmol/L)	11.182±5.400	15.544±10.584	13.386±10.076	9.676±3.248	13.557±9.931	11.099±4.983
Folate (ng/ml)	7.862±6.730	6.272±7.552	7.820±4.012	8.573±5.008	7.724±5.387	10.034±7.302
Correlation coefficient [a]	-0.208	-0.090	-0.149	-0.214	-0.084	-0.130
P value	0.037	0.251	0.179	0.030	0.334	0.199
***RFC-1* –43TT genotype**						
tHcy (µmol/L)	9.565±2.228	15.215±10.042	11.495±6.231	10.239±4.452	13.563±13.879	9.885±2.237
Folate (ng/ml)	8.567±5.083	6.138±6.637	8.227±5.344	9.157±4.893	7.882±5.391	8.908±4.808
Correlation coefficient [a]	-0.086	-0.058	-0.320	-0.480	-0.120	-0.353
P value	0.582	0.626	0.104	0.010	0.360	0.041

***RFC-1* 80A>G**

Characteristics	Male Control (n=262)	Male Ischemic stroke (n=328)	Male SBI (n=163)	Female Control (n=243)	Female Ischemic stroke (n=256)	Female SBI (n=190)
***RFC-1* 80AA genotype**						
tHcy (µmol/L)	10.096±2.905	16.092±11.633	12.329±3.756	9.736±3.308	14.096±11.001	10.733±4.708
Folate (ng/ml)	8.216±6.321	6.721±8.826	9.098±7.735	11.460±13.395	7.923±5.242	9.329±5.831
Correlation coefficient [a]	0.101	-0.061	-0.039	-0.151	0.011	-0.209
P value	0.444	0.550	0.820	0.172	0.925	0.122
***RFC-1* 80AG genotype**						
tHcy (µmol/L)	11.365±5.517	15.760±11.087	13.238±10.108	9.723±3.376	13.811±10.209	11.118±4.972
Folate (ng/ml)	7.469±6.783	6.265±6.141	7.737±3.733	8.888±5.733	7.847±5.474	10.010±7.287
Correlation coefficient [a]	-0.197	-0.104	-0.167	-0.215	-0.089	-0.126
P value	0.058	0.206	0.137	0.043	0.326	0.214
***RFC-1* 80GG genotype**						
tHcy (µmol/L)	9.416±2.212	15.735±10.534	11.766±5.996	10.196±4.356	13.575±13.987	9.797±2.263
Folate (ng/ml)	8.898±5.223	6.147±6.612	8.023±5.066	9.362±4.696	7.579±5.361	8.999±4.767
Correlation coefficient [a]	-0.105	-0.064	-0.350	-0.499	-0.101	-0.367
P value	0.521	0.588	0.043	0.005	0.448	0.030

***RFC-1* 696T>C**

Characteristics	Male Control (n=262)	Male Ischemic stroke (n=328)	Male SBI (n=163)	Female Control (n=243)	Female Ischemic stroke (n=256)	Female SBI (n=190)
***RFC-1* 696TT genotype**						
tHcy (µmol/L)	10.208±3.019	17.049±12.642	12.266±3.770	9.904±3.501	15.066±11.930	10.659±4.667
Folate (ng/ml)	7.760±6.513	6.667±6.809	8.853±7.543	12.545±14.541	7.750±5.505	9.024±4.966
Correlation coefficient [a]	0.114	-0.063	-0.078	-0.174	0.041	-0.386
P value	0.425	0.574	0.649	0.147	0.758	0.005
***RFC-1* 696TC genotype**						
tHcy (µmol/L)	11.228±5.373	15.375±10.531	13.201±9.942	9.525±3.240	13.569±9.919	11.089±5.023
Folate (ng/ml)	7.885±6.813	6.426±7.672	7.887±4.013	8.596±5.032	7.871±5.374	10.277±7.576
Correlation coefficient [a]	-0.202	-0.093	-0.146	-0.205	-0.092	-0.082
P value	0.044	0.237	0.187	0.040	0.291	0.414
***RFC-1* 696CC genotype**						
tHcy (µmol/L)	9.453±2.300	15.590±10.558	11.820±6.171	10.525±4.280	13.281±13.596	10.012±2.329
Folate (ng/ml)	8.510±4.952	5.973±6.407	7.975±5.122	8.890±4.816	7.720±5.299	8.678±4.678
Correlation coefficient [a]	-0.127	-0.069	-0.351	-0.486	-0.111	-0.370
P value	0.419	0.548	0.049	0.006	0.385	0.024

SBI indicates silent brain infarction.

[a] Correlation coefficient using the Pearson correlation analysis.

Table S8. Plasma homocysteine levels and variability among *RFC-1* -43C>T, 80A>G, and 696T>C genotypes in ischemic stroke, ischemic-stroke subtype, silent brain infarction (SBI), and control subjects

Group	Mean±SD (n)	CV, %	Mean±SD (n)	CV, %	Mean±SD (n)	CV, %	P^a
***RFC-1* –43C>T**	CC		CT		TT		
Controls	9.984±3.249 (145)	32.5	10.430±4.519 (262)	43.3	9.811±3.216 (93)	32.8	0.327
Ischemic stroke	11.280±6.655 (148)	58.9	11.570±5.659 (303)	48.9	11.840±8.916 (133)	75.3	0.785
Small-artery occlusion (SAO)	11.260±7.753 (37)	68.9	11.320±4.218 (77)	37.3	10.920±5.740 (45)	52.6	0.930
Large-artery occlusion (LAO)	11.060±5.975 (64)	54.0	12.100±7.122 (128)	58.9	11.680±5.732 (49)	49.1	0.588
Cardio embolism	12.330±7.978 (17)	64.7	10.240±3.714 (36)	36.3	10.990±3.987 (17)	36.3	0.385
Undetermined	11.150±6.143 (29)	55.1	11.880±4.610 (57)	38.8	14.750±18.23 (22)	123.6	0.364
SBI	11.330±4.308 (102)	38.0	12.160±7.838 (185)	64.5	10.640±4.599 (64)	43.2	0.227
***RFC-1* 80A>G**	AA		AG		GG		
Controls	9.902±3.124 (171)	31.5	10.580±4.676 (237)	44.2	9.721±3.225 (92)	33.2	0.109
Ischemic stroke	11.510±6.604 (172)	57.4	11.440±5.615 (279)	49.1	11.860±8.902 (133)	75.1	0.841
Small-artery occlusion (SAO)	11.540±7.752 (40)	67.2	11.130±4.041 (74)	36.3	10.990±5.697 (45)	51.8	0.896
Large-artery occlusion (LAO)	11.270±5.957 (75)	52.9	12.050±7.215 (118)	59.9	11.700±5.778 (48)	49.4	0.724
Cardio embolism	11.730±7.121 (22)	60.7	10.310±3.939 (30)	38.2	10.980±3.951 (18)	36.0	0.619
Undetermined	11.850±6.544 (34)	55.2	11.530±4.089 (52)	35.5	14.670±18.23 (22)	124.3	0.404
SBI	11.400±4.387 (96)	38.5	12.100±7.827 (184)	64.7	10.800±4.631 (71)	42.9	0.323
***RFC-1* 696T>C**	TT		TC		CC		
Controls	10.050±3.276 (145)	32.6	10.390±4.522 (259)	43.5	9.866±3.232 (96)	32.8	0.481
Ischemic stroke	11.590±6.965 (142)	60.1	11.470±5.568 (300)	48.5	11.700±8.665 (142)	74.1	0.944
Small-artery occlusion (SAO)	11.620±8.192 (35)	70.5	11.150±4.021 (79)	36.1	10.940±5.678 (45)	51.9	0.862
Large-artery occlusion (LAO)	11.120±6.080 (60)	54.7	12.120±7.166 (126)	59.1	11.540±5.562 (55)	48.2	0.609
Cardio embolism	12.540±7.868 (17)	62.7	10.200±3.840 (33)	37.6	10.760±3.851 (20)	35.8	0.312
Undetermined	11.960±6.954 (29)	58.1	11.590±4.033 (57)	34.8	14.530±18.25 (22)	125.6	0.445
SBI	11.350±4.357 (91)	38.4	12.070±7.764 (189)	64.3	10.880±4.644 (71)	42.7	0.366

CV; between-person coefficient of variations.

[a] Kruskal-Wallis non-parametric test of plasma homocysteine levels among genotypes.

Table S9. Plasma folate levels and variability among *RFC-1* -43C>T, 80A>G, and 696T>C genotypes in ischemic stroke, ischemic-stroke subtype, silent brain infarction (SBI), and control subjects

Group	Mean±SD (n)	CV, %	Mean±SD (n)	CV, %	Mean±SD (n)	CV, %	P^a
***RFC-1* –43C>T**	CC		CT		TT		
Controls	10.520±12.070 (122)	114.7	8.217±5.928 (206)	72.1	8.800±4.982 (71)	56.6	0.049
Ischemic stroke	7.251±6.267 (147)	86.4	6.923±6.698 (299)	96.7	6.925±6.146 (133)	88.8	0.869
Small-artery occlusion (SAO)	6.592±4.168 (37)	63.2	7.464±7.060 (77)	94.6	6.313±3.279 (45)	51.9	0.503
Large-artery occlusion (LAO)	6.400±4.388 (63)	68.6	5.994±3.902 (127)	65.1	7.057±7.074 (49)	100.2	0.466
Cardio embolism	11.500±10.510 (17)	91.4	8.777±12.800 (34)	145.8	6.440±3.347 (17)	52.0	0.387
Undetermined	7.591±7.931 (29)	104.5	6.720±5.085 (56)	75.7	8.256±7.382 (22)	89.4	0.610
SBI	9.059±6.356 (100)	70.2	9.024±6.114 (182)	67.8	8.601±5.026 (62)	58.4	0.874
***RFC-1* 80A>G**	AA		AG		GG		
Controls	10.110±11.100 (144)	109.8	8.152±6.323 (185)	77.6	9.097±4.974 (70)	54.7	0.101
Ischemic stroke	7.223±7.543 (170)	104.4	6.981±5.891 (276)	84.4	6.782±6.109 (133)	90.1	0.838
Small-artery occlusion (SAO)	6.439±4.075 (40)	63.3	7.617±7.166 (74)	94.1	6.253±3.262 (45)	52.2	0.354
Large-artery occlusion (LAO)	6.496±4.379 (74)	67.4	5.941±3.928 (117)	66.1	6.970±8.061 (48)	115.7	0.476
Cardio embolism	11.52±15.890 (22)	137.9	8.590±8.147 (28)	94.8	6.081±3.226 (18)	53.1	0.273
Undetermined	7.063±7.464 (33)	105.7	7.084±5.241 (52)	74.0	8.030±7.462 (22)	92.9	0.827
SBI	9.236±6.628 (94)	71.8	8.987±6.049 (180)	67.3	8.511±4.908 (70)	57.7	0.744
***RFC-1* 696T>C**	TT		TC		CC		
Controls	10.540±12.060 (122)	114.4	8.239±5.989 (203)	72.7	8.669±4.865 (74)	56.1	0.046
Ischemic stroke	7.113±6.307 (141)	88.7	7.080±6.757 (296)	95.4	6.748±5.985 (142)	88.7	0.860
Small-artery occlusion (SAO)	6.438±4.240 (35)	65.9	7.514±6.976 (79)	92.8	6.304±3.267 (45)	51.8	0.430
Large-artery occlusion (LAO)	6.321±4.187 (59)	66.2	6.120±4.085 (125)	66.7	6.771±7.674 (55)	113.3	0.737
Cardio embolism	11.030±10.730 (17)	94.0	9.403±13.300 (31)	141.4	6.219±3.144 (20)	50.6	0.369
Undetermined	7.375±7.943 (29)	107.7	6.900±5.083 (56)	73.7	8.083±7.428 (22)	91.9	0.764
SBI	8.953±6.130 (89)	68.5	9.192±6.315 (185)	68.7	8.347±4.869 (70)	58.3	0.605

CV; between-person coefficient of variations.

[a] Kruskal-Wallis non-parametric test of plasma folate levels among genotypes.

Table S10. AOR values of ischemic stroke and silent brain infarction (SBI) prevalence among *RFC-1* genotypes in samples recruited from 2004 to 2007

Genotype	Control (%) n=269	Ischemic stroke Case (%) n=310	AOR (95% CI)[a]	*P*	*P*[c]	Silent brain infarction Case (%) n=230	AOR (95% CI)[b]	*P*	*P*[c]
***RFC-1* -43C>T**									
CC	78 (29.0)	67 (21.6)	1.000 (reference)			65 (28.3)	1.000 (reference)		
CT	141 (52.4)	164 (52.9)	1.417 (0.922 - 2.178)	0.112	0.168	120 (52.2)	0.991 (0.654 - 1.502)	0.968	0.968
TT	50 (18.6)	79 (25.5)	2.003 (1.183 - 3.394)	0.010	0.010	45 (19.6)	1.082 (0.638 - 1.833)	0.771	0.771
CC vs. CT+TT (Dominant)			1.560 (1.035 - 2.353)	0.034	0.051		1.007 (0.678 - 1.496)	0.974	0.974
CC+CT vs. TT (Recessive)			1.530 (0.993 - 2.358)	0.054	0.054		1.084 (0.688 - 1.710)	0.727	0.727
***RFC-1* 80A>G**									
AA	88 (32.7)	89 (28.7)	1.000 (reference)			61 (26.5)	1.000 (reference)		
AG	132 (49.1)	141 (45.5)	1.087 (0.723 - 1.635)	0.689	0.689	118 (51.3)	1.261 (0.833 - 1.910)	0.273	0.417
GG	49 (18.2)	80 (25.8)	1.699 (1.025 - 2.816)	0.040	0.040	51 (22.2)	1.522 (0.904 - 2.563)	0.115	0.333
AA vs. AG+GG (Dominant)			1.235 (0.842 - 1.813)	0.280	0.280		1.319 (0.890 - 1.955)	0.168	0.338
AA+AG vs. GG (Recessive)			1.581 (1.024 - 2.442)	0.039	0.054		1.289 (0.824 - 2.016)	0.266	0.671
***RFC-1* 696T>C**									
TT	80 (29.7)	60 (19.4)	1.000 (reference)			56 (24.3)	1.000 (reference)		
TC	139 (51.7)	166 (53.5)	1.630 (1.053 - 2.524)	0.028	0.084	126 (54.8)	1.264 (0.828 - 1.930)	0.278	0.417
CC	50 (18.6)	84 (27.1)	2.533 (1.486 - 4.316)	0.001	0.003	48 (20.9)	1.390 (0.819 - 2.361)	0.222	0.333
TT vs. TC+CC (Dominant)			1.853 (1.218 - 2.819)	0.004	0.012		1.285 (0.858 - 1.925)	0.225	0.338
TT+TC vs. CC (Recessive)			1.720 (1.120 - 2.639)	0.013	0.039		1.191 (0.759 - 1.868)	0.447	0.671

[a] Adjusted by age, gender, hypertension, diabetes mellitus, hyperlipidemia, and smoking.

[b] Adjusted by age, gender, hypertension, diabetes mellitus, and hyperlipidemia.

[c] False positive discovery rate-adjusted *P*-value.

Table S11. AOR values of ischemic stroke and silent brain infarction (SBI) prevalence among *RFC-1* genotypes in samples recruited from 2008 to 2010

Genotype	Control (%) n=236	Ischemic stroke Case (%) n=274	AOR (95% CI)[a]	*P*	*P*[c]	Silent brain infarction Case (%) n=123	AOR (95% CI)[b]	*P*	*P*[c]
***RFC-1* -43C>T**									
CC	68 (28.8)	81 (29.6)	1.000 (reference)			38 (30.9)	1.000 (reference)		
CT	124 (52.5)	139 (50.7)	1.045 (0.677 - 1.613)	0.841	0.891	65 (52.8)	0.917 (0.546 - 1.541)	0.744	0.797
TT	44 (18.6)	54 (19.7)	1.062 (0.621 - 1.817)	0.827	0.999	20 (16.3)	0.725 (0.352 - 1.492)	0.383	0.785
CC vs. CT+TT (Dominant)			1.061 (0.707 - 1.592)	0.775	0.959		0.909 (0.556 - 1.487)	0.704	0.833
CC+CT vs. TT (Recessive)			1.086 (0.681 - 1.730)	0.730	0.773		0.876 (0.480 - 1.600)	0.667	0.927
***RFC-1* 80A>G**									
AA	84 (35.6)	83 (30.3)	1.000 (reference)			36 (29.3)	1.000 (reference)		
AG	108 (45.8)	138 (50.4)	1.421 (0.930 - 2.171)	0.104	0.312	66 (53.7)	1.485 (0.885 - 2.490)	0.134	0.402
GG	44 (18.6)	53 (19.3)	1.267 (0.749 - 2.145)	0.377	0.999	21 (17.1)	1.041 (0.528 - 2.051)	0.908	0.908
AA vs. AG+GG (Dominant)			1.379 (0.931 - 2.042)	0.109	0.327		1.382 (0.850 - 2.247)	0.192	0.576
AA+AG vs. GG (Recessive)			1.071 (0.672 - 1.706)	0.773	0.773		0.889 (0.493 - 1.605)	0.696	0.927
***RFC-1* 696T>C**									
TT	66 (28.0)	82 (29.9)	1.000 (reference)			36 (29.3)	1.000 (reference)		
TC	123 (52.1)	134 (48.9)	0.970 (0.626 - 1.503)	0.891	0.891	63 (51.2)	0.932 (0.548 - 1.587)	0.797	0.797
CC	47 (19.9)	58 (21.2)	1.000 (0.592 - 1.689)	0.999	0.999	24 (19.5)	0.798 (0.398 - 1.598)	0.523	0.785
TT vs. TC+CC (Dominant)			0.989 (0.659 - 1.486)	0.959	0.959		0.948 (0.575 - 1.562)	0.833	0.833
TT+TC vs. CC (Recessive)			1.080 (0.687 - 1.699)	0.739	0.773		0.974 (0.550 - 1.723)	0.927	0.927

[a] Adjusted by age, gender, hypertension, diabetes mellitus, hyperlipidemia, and smoking.

[b] Adjusted by age, gender, hypertension, diabetes mellitus, and hyperlipidemia.

[c] False positive discovery rate-adjusted *P*-value.

Table S12. AOR values of small-artery occlusion prevalence among *RFC-1* genotypes in samples recruited from 2004 to 2007

Genotype	Control (%) n=269	Case (%) n=77	AOR (95% CI)[a]	*P*	*P*[b]
***RFC-1* -43C>T**					
CC	78 (29.0)	18 (23.4)	1.000 (reference)		
CT	141 (52.4)	35 (45.5)	1.122 (0.573 - 2.197)	0.737	0.737
TT	50 (18.6)	24 (31.2)	2.412 (1.115 - 5.220)	0.025	0.025
CC vs. CT+TT (Dominant)			1.440 (0.769 - 2.697)	0.255	0.255
CC+CT vs. TT (Recessive)			2.085 (1.122 - 3.872)	0.020	0.020
***RFC-1* 80A>G**					
AA	88 (32.7)	20 (26.0)	1.000 (reference)		
AG	132 (49.1)	33 (42.9)	1.177 (0.613 - 2.259)	0.624	0.737
GG	49 (18.2)	24 (31.2)	2.567 (1.177 - 5.597)	0.018	0.025
AA vs. AG+GG (Dominant)			1.488 (0.814 - 2.720)	0.197	0.255
AA+AG vs. GG (Recessive)			2.123 (1.133 - 3.979)	0.019	0.020
***RFC-1* 696T>C**					
TT	80 (29.7)	15 (19.5)	1.000 (reference)		
TC	139 (51.7)	38 (49.4)	1.508 (0.750 - 3.033)	0.249	0.737
CC	50 (18.6)	24 (31.2)	3.443 (1.511 - 7.844)	0.003	0.009
TT vs. TC+CC (Dominant)			1.913 (0.986 - 3.711)	0.055	0.165
TT+TC vs. CC (Recessive)			2.244 (1.205 - 4.178)	0.011	0.020

[a] Adjusted by age, gender, hypertension, diabetes mellitus, hyperlipidemia, and smoking.
[b] False positive discovery rate-adjusted *P*-value.

Table S13. AOR values of small-artery occlusion prevalence among *RFC-1* genotypes in samples recruited from 2008 to 2010

Genotype	Control (%) n=236	Case (%) n=82	AOR (95% CI)[a]	*P*	*P*[b]
***RFC-1* -43C>T**					
CC	68 (28.8)	19 (23.2)	1.000 (reference)		
CT	124 (52.5)	42 (51.2)	1.518 (0.791 - 2.911)	0.209	0.314
TT	44 (18.6)	21 (25.6)	1.932 (0.892 - 4.182)	0.095	0.143
CC vs. CT+TT (Dominant)			1.640 (0.891 - 3.019)	0.112	0.168
CC+CT vs. TT (Recessive)			1.556 (0.837 - 2.892)	0.162	0.243
***RFC-1* 80A>G**					
AA	84 (35.6)	20 (24.4)	1.000 (reference)		
AG	108 (45.8)	41 (50.0)	1.885 (1.000 - 3.552)	0.050	0.150
GG	44 (18.6)	21 (25.6)	2.089 (0.988 - 4.420)	0.054	0.143
AA vs. AG+GG (Dominant)			1.971 (1.091 - 3.561)	0.025	0.075
AA+AG vs. GG (Recessive)			1.566 (0.847 - 2.893)	0.153	0.243
***RFC-1* 696T>C**					
TT	66 (28.0)	20 (24.4)	1.000 (reference)		
TC	123 (52.1)	41 (50.0)	1.380 (0.721 - 2.644)	0.331	0.331
CC	47 (19.9)	21 (25.6)	1.581 (0.744 - 3.360)	0.233	0.233
TT vs. TC+CC (Dominant)			1.453 (0.794 - 2.659)	0.225	0.225
TT+TC vs. CC (Recessive)			1.431 (0.777 - 2.637)	0.250	0.250

[a] Adjusted by age, gender, hypertension, diabetes mellitus, hyperlipidemia, and smoking.
[b] False positive discovery rate-adjusted *P*-value.
